# The MoVIN server for the analysis of protein interaction networks

**DOI:** 10.1186/1471-2105-9-S2-S11

**Published:** 2008-03-26

**Authors:** Paolo Marcatili, Giovanni Bussotti, Anna Tramontano

**Affiliations:** 1 Department of Biochemical Sciences, “Sapienza” University, Rome, Italy; 2 Istituto Pasteur – Fondazione “Cenci Bolognetti”, Rome, Italy

## Abstract

**Background:**

Protein-protein interactions are at the basis of most cellular processes and crucial for many bio-technological applications. During the last few years the development of high-throughput technologies has produced several large-scale protein-protein interaction data sets for various organisms. It is important to develop tools for dissecting their content and analyse the information they embed by data-integration and computational methods.

**Results:**

Interactions can be mediated by the presence of specific features, such as motifs, surface patches and domains. The co-occurrence of these features on proteins interacting with the same protein can indicate mutually exclusive interactions and, therefore, can be used for inferring the involvement of the proteins in common biological processes.

We present here a publicly available server that allows the user to investigate protein interaction data in light of other biological information, such as their sequences, presence of specific domains, process and component ontologies. The server can be effectively used to construct a high-confidence set of mutually exclusive interactions by identifying similar features in groups of proteins sharing a common interaction partner. As an example, we describe here the identification of common motifs, function, cellular localization and domains in different datasets of yeast interactions.

**Conclusions:**

The server can be used to analyse user-supplied datasets, it contains pre-processed data for four yeast Protein Protein interaction datasets and the results of their statistical analysis. These show that the presence of common motifs in proteins interacting with the same partner is a valuable source of information, it can be used to investigate the properties of the interacting proteins and provides information that can be effectively integrated with other sources. As more experimental interaction data become available, this tool will become more and more useful to gain a more detailed picture of the interactome.

## Background

Protein functions are mediated and regulated through a complex network of interactions [1]. In many cases proteins physically bind to each other to absolve their role, and the interaction is often mediated by the physical binding of some of their subunits, such as domains, surface patches or small regions composed of a few residues called motifs [2-4]. Although the latter is rather frequent, there have been few attempts to systematically explore the information that they provide at the genomic level. Motif recognition has proven to be very useful in many biological contexts, but is not an easy task [3,5,6]. Motifs are often short in length (three to twelve residues), they are often located in disordered regions of the proteins and their conservation is limited to closely related species. Nevertheless the identification of shared motifs has proven to be very useful to characterize protein interactions (e.g. the binding of the SH3 domain to the PxxP local sequence), function (DNA binding), localization (nuclear localization signal) and domain fingerprints (PROSITE [4]).

The recent development of high-throughput technologies for detecting protein-protein interactions (PPIs) has produced many publicly available databases [1,7-10]. Although the accuracy of the data is not always optimal [11,12], the information they provide is of primary importance for formulating biologically relevant hypotheses and it is therefore essential to develop tools for analysing and dissecting them. There are methods that make use of different biological data to assess the reliability of interactions: gene expression [13], homology [14], Gene Ontology (GO) annotations [15], phylogenetic features [16], synthetic lethality, domain interaction [17], and a combination of these [18]. PPI maps have also been mined to infer functional similarity, domain interactions and protein motifs [5,19,20].

In this work we describe a server for simplifying the analysis of the features shared by proteins interacting with the same partner. We show here its power by investigating the presence of sequence motifs in yeast PPI maps and their correlation with the presence of similar Gene Ontology annotations (process and component) [15] and Pfam domains [21]. The result of our analysis is that the information that can be gained by motif detection is relevant and coherent with functional, localization and domain data but it is not redundant with respect to these other sources of information. It is indeed possible to exploit the presence of common motifs to identify mutually exclusive interactions and to estimate the reliability of a PPI map.

## Results and discussion

### The MoVIN server

**Figure 1:**
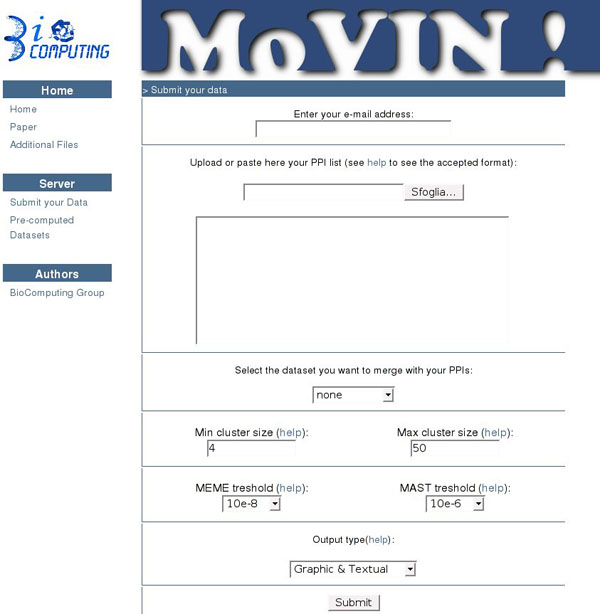
**Snapshot of the input page of the MoVIN server**. The user can upload a Protein-protein interaction map in any of the accepted formats (tab or comma separated) with or without merging it with existing datasets. The minimum and maximum cluster size as well as the threshold E-values for MEME and MAST can be selected as well.

The MoVIN server can load experimental PPI datasets and perform an analysis of sets of interactions sharing a common partner (Figure 1). It contains pre-computed data for four dataset for S. cerevisiae of different size, level of curation and estimated false positive rate, which overlap only partially (see Methods).

Given a dataset, the tool automatically extracts the groups of proteins sharing a common interaction partner (see Methods). These are the sets of all and only the proteins that bind to a common protein partner. This central protein (hub) is used to identify the cluster. The user can select the minimum size of the cluster. For each cluster, MoVIN collates the corresponding set of protein sequences and searches them for the presence of common motifs using MEME [22] and MAST [23].

MEME (vers. 3.5.3) is a tool for discovering motifs in a group of related DNA or protein sequences. MEME takes as input a group of sequences (in this case the sequences of the proteins in a cluster) and outputs as many motifs as possible according to the constraints given by the user. MEME uses statistical modelling techniques to automatically choose the best length and description for each motif. In MoVIN we use as background distribution the average composition of the complete set of proteins in the map and focus on motifs with length between three and twelve residues and with an E-value (as reported by the output of the program) lower than a user-defined threshold (default is 10e-7). To avoid that motifs with a strong signal (e.g. fingerprints of a domain shared by some proteins of the group) mask “weaker” motifs, the server repeats this step on each cluster many times, recursively eliminating the proteins for which a common motif has already been identified.

In order to estimate the specificity of the motifs found by MEME, they are mapped on all the proteins in the dataset using MAST (vers. 3.5.3). For each motif, MAST returns a list of proteins containing it, the position of the match in the sequence and the corresponding E-value. Only matches with an E-value smaller than a user-defined threshold (default is 10e-5, i.e. 100 times larger than the E-value threshold chosen for MEME, in order to prevent overfitting of the motifs on the initial set of clustered proteins) are retained.

Next, the server assigns to each cluster C_i_ and each motif M_j_ a *motif P value* S_i,j_ related to the over-representation of the motif in that cluster. If the motif has been found on x_ij_ protein in the cluster C_i_ of n_i_ proteins and on X_j_ proteins in the complete dataset of size N we have S^m^_i,j_=hgcdf(x_ij,_ n_i_, X_j_, N) where hgcdf is the hypergeometric cumulative distribution function, which measures the probability of finding at least as many occurrences of the motif in a cluster of similar size randomly extracted from the whole set of proteins.

Finally, the previously calculated scores are assigned to the interaction between the central protein of the cluster and each protein in the cluster containing the motif M_j_. Different motifs can be present on a protein and one protein of a cluster can be, at the same time, the hub protein of another cluster, therefore different scores can be associated to the same interaction. In such cases, we assign the minimum score to the interaction.

The *process P value* S^p^, the *component P value* S^l^ and the *domain P value* S^d^ are computed in a similar fashion. The GO process score and the GO component score are calculated by mapping the corresponding GO ontology terms on each protein with the program BatchGoViewer, which returns, given a list of proteins, the annotations (at any level) with the lowest P values. The Pfam domain score is calculated by analysing the presence of Pfam A Domains on each protein. The protein-domain relationship is taken from the Ensembl website [24].

The results of all the analyses are displayed with a user-friendly interface, including textual search and graphics tools. The cluster and their features are graphically visualised using GraphViz. For each displayed item there are links to several different publicly available databases.

Additionally, the user can look for the presence of known mutations and for their position in the highlighted proteins (as reported in the Protein Mutant Database) [25]. It is possible to download and visualize experimental structures or three-dimensional models for a large fraction of the yeast genome. Known structures are downloaded from the Protein Data Bank [26], models are downloaded from the ModBase database [27]. Each structure or model can be visualised in the web browser via Jmol [28] and all the motifs found by the MoVIN web server can be highlighted.

### Application of the MoVIN server to the S.cerevisiae interactome

We used MoVIN to analyse the BioGRID, BIND, Gavin06 and Krogan datasets (Table 1). We only considered clusters containing more than four proteins and used the default values for all the parameters. A summary of the final datasets is reported in Table 1.

**Table 1:** Dataset Summary. The number of interactions in each dataset is between 942 (Uetz) and 51,086 (BioGRID), the number of clusters containing more than 4 proteins is between 100 (Uetz) and 3,963. The average number of proteins in each cluster ranges from 6.15 (Uetz) to 24.96 (BioGRID).

Dataset	# of interactions	# of clusters	average cluster size
BIND	8847	1271	9.60
BioGRID	51086	3963	24.96
Gavin02	3500	522	9.78
Gavin06	19973	1738	20.94
Krogan	6699	1042	10.44
Uetz	942	100	6.15

First of all, we analysed the motif P value distribution generated by the server using as input the original datasets and then compared them with the corresponding background distribution. Such background distribution is obtained -for each PPI map- by randomizing the set of interactions of the original map (written as ordered pairs) with no duplicate (i.e. if the pair (a_j_, b_j_) is present we can not have the pair (b_j_, a_j_)). We randomly shuffle all the second terms of each pair and remove duplicate interactions. By doing so, we preserve the connectivity degree of each protein and hub proteins remain such in the randomized map. The total number of interactions of each randomized map differs by less than 1% from that of the original map.

**Figure 2:**
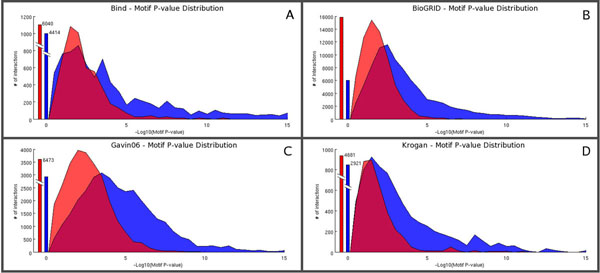
**Motif P Value Distributions for yeast datasets.** We report the number of interactions with Motif P value lower than the threshold (reported on the × axis in logarithmic scale) for the experimental datasets (blue) and for its randomized version (red). The interactions for which no motif was found are reported as bars in the origin. The Motif P value distribution for the experimental datasets contains a larger fraction of interactions with respect to the random datasets and is shifted towards lower P values. (A) BIND, (B) BioGRID, (C) Gavin06 and (D) Krogan datasets.

The difference between the original dataset P value distributions and their randomized version is computed by a permutation test on the means. We generate a global distribution by merging the original and the randomized dataset distribution. Next, we randomly generate 100.000 distributions of the same size as the original dataset and compute their means. The distribution of the means for the analysed datasets is normal (D'Agostino-Pearson test, significance=0.05), therefore we can compute the standard Z-score of our original dataset:

$$z=\frac{\left(M_{d}-M\right)}{\sigma }$$

where *M_d_* is the mean of the distribution on the original dataset, *M* is the mean and σ is the standard deviation of the mean distribution. The randomization step was repeated 5 times for each dataset, yielding each time similar results (data not shown). The results (Figure 2 and Table 2) show that the motif distributions in PPI maps are far from random.

**Table 2:** Statistical Significance of the Motif P value Distributions. The table reports the Z-score of the mean of the experimental distribution with respect to the random distribution of the means. The latter was obtained by computing the means of 100,000 distributions of the same size of the experimental one obtained by randomly extracting interactions from the original and the randomized distributions.

BIND	Motif	Process	Component	Domain
33,17	127,70	26,46	82,85	32,31

It is interesting to note that several parameters, such as the percentage of interactions that can be explained by a motif on one of the binding partners or the difference between the occurrence of the motifs in the real map and in the randomized one are consistent with the expected fraction of spurious interaction present in the datasets. As shown in Table 2, MoVIN finds more motifs in the databases BioGRID (that is the largest available repository of PPIs) and BIND (that only contains well annotated PPIs and is manually-curated), while it finds far less common motifs in the Uetz dataset (which is estimated to contain a relatively larger fraction of false positives [11]). Moreover, the effectiveness of the method increases with the dimension of the map. The more the map covers the complete interactome, the larger the number of motifs identified.

On the basis of this analysis, we generated a high-confidence subset of the yeast PPI datasets (see additional files 1 and 2) that contains 17.733 interactions (see Methods).

Such interactions, as previously stated, are likely to be mutually exclusive.

Although the motifs that we identify are not necessarily related to physical binding of the proteins (they could be functional motifs or localization signals) and the motif-selected dataset is biased towards mutually exclusive interactions, it is likely that our selected subset is enriched in true positive interactions, and can be a good candidate for applications that need a benchmark interaction dataset.

### Comparison with GO annotations and Pfam domains

The MoVIN server can also use other sources of information commonly used to assess the reliability of interaction data, i.e. GO annotations (process and component) and Pfam domains. An important question is to which extent the motif information overlaps with that given by the co-occurrence of GO terms and the presence of similar Pfam domain in proteins interacting with a common partner.

To address the issue, we applied the method to the same yeast datasets described above. Here we only report the results for the BIND dataset, the results for the other datasets are very similar (data not shown).

The correlations between log_10_ (Motif P value) and the P values for GO process, GO component and Pfam (Table 3) are positive in all cases and range from 0.25 (motif vs component P value) to 0.56 (motif vs domain P value). This indicates that the information obtained from the motif analysis is consistent, but not completely redundant, with the other sources of information here considered.

**Table 3:** Correlation coefficient between Motif, Process, Component and Domain P values

BIND	Motif	Process	Component
Motif	1.00	0.30	0.26
Process	0.30	1.00	0.80
Component	0.26	0.80	1.00
Domain	0.57	0.40	0.37

### An example: function prediction using motif analysis

The method that we described for the analysis of PPI maps can almost naturally be extended to become a tool for protein functional assignment, on the basis of the hypothesis that two proteins interacting with the same partner and sharing a common motif are likely to have some functional similarity as well. Here we will just describe one instructive example of the potential of the approach, both in assigning functions and in detecting erroneous or outdated annotations.

**Figure 3:**
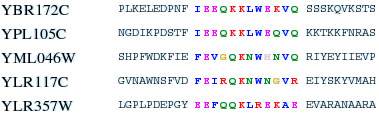
**Alignment of the motif present in 5 out of the 7 proteins binding to YKL074C.** The first protein (YBR172C) is annotated as “Cytoskeleton organization and biogenesis”, the second (YPL105C) does not have any GO annotation. The last three (YML046W, YLR117C and YLR357W) are annotated with terms related to the spliceosome activity.

We found a motif shared by 5 among 7 proteins in the YKL074C cluster (BIND dataset). An alignment of the sequences is reported in Figure 3. The motif displays several conserved residues and does not match any pattern found in the PROSITE database [4].

The hub protein YKL074C and 4 proteins in the group (YDL043C, YML046W and YLR117C, where the latter two contain the motif) have a GO process annotation that is related to the spliceosome. Another protein (YLR357W) has an annotation (double-strand break repair via nonhomologous end joining) which is consistent with the splicing pathway. The two proteins YBR172C and YPL105C are homologous and both contain the motif. The second protein is not annotated, while the first has a GO process annotation “Cytoskeleton organization and biogenesis”. Interestingly, more recent experimental evidence supports the hypothesis of its involvement with RNA splicing [29]. It is reasonable to suggest that YPL105C should be tentatively annotated as involved in RNA splicing and that the GO annotation for YBR172C should be verified.

## Conclusions

We have described here a tool for the analysis of protein interaction maps, able to correlate the co-occurrence of sequence motifs, common GO annotations and similar Pfam domains with interactions sharing a common partner. Furthermore, we have investigated the relationship between the presence of common motifs and the presence of shared functional, component and domain data. The information given by the presence of common motifs is coherent and complementary to that present in other data sources. All these sources could be integrated to generate a large high-confidence yeast PPI dataset.

As further developments of the server we are extending the approach to the search for discontinuous motifs brought together by the three-dimensional structures of the interacting proteins.

## Methods

### Data sources

We analysed yeast (Saccharomyces Cerevisiae) PPI maps obtained from different datasets: the databases BIND [7] and BioGRID [9], the experimental datasets Gavin02 [30], Gavin06 [31], Krogan [32] and Uetz [33]. We filtered out the data for which it was not possible to precisely identify both interacting partner (e.g. multi-protein complexes).

The BIND dataset contains 8.847 yeast interactions, manually curated and annotated. BioGRID contains 51.086 interactions extracted from the literature and obtained with different experimental methods. The datasets from Gavin and Krogan are from three different tandem affinity purification (TAP) experiments and contain respectively 3.500 (Gavin02), 19.973 (Gavin06) and 6.699 (Krogan) interactions. The Uetz dataset contains 942 interactions derived from a Yeast 2 Hybrid experiment.

Protein sequences were downloaded from the Ensembl Genome Browser website on July 7^th^ 2006).

To assign the GO terms, we used the annotation files downloaded from the GO website and only used IDA (Inferred from Direct Assay), IGI (Inferred from Genetic Interaction), IMP (Inferred from Mutant Phenotype) and TAS (Traceable Author Statement) annotations as to avoid low-confidence or indirect annotations.

The high-confidence PPI dataset (MOVIN_1) was generated by merging all the interactions that, at least in one dataset, were reported to have a motif P value smaller than 10e-4. Such dataset contains 17.733 interactions and can be downloaded from 

A second high-confidence PPI dataset (MOVIN_2) was generated by merging all the interactions that, at least in one dataset, were reported to have a significant sequence motif and at least a GO function or a GO component P value smaller than 10e-4. This dataset contains 14.103 interactions and can be downloaded from 

## Competing interests

The authors declare that they have no competing interests.

## Authors' contributions

AT conceived the study, participated in its design and coordination and helped to draft the manuscript. GB helped in data analysis. PM developed the method, implemented it and performed the statistical analysis. All authors read and approved the final manuscript

## Supplementary Material

Additional file 1 – MOVIN1.list List of all interactions between proteins interacting with the same partner and containing a motif with a P value <10e-4. (CSV file). Click here for file

Additional file 2 – MOVIN2.list List of all the interactions between proteins interacting with the same partner, containing a motif with a P value <10e-4 and sharing a process or component GO term with a P value <10e-4. (CSV file). Click here for file
